# The Purity of Milk

**Published:** 1898-12-03

**Authors:** 


### THE PURITY OF MILK.

ISOLA writes: I should esteem it a great favour if you will kindly inform me of a cheap but sure test for proving the purity (or otherwise) of milk.

*** Unfortunately the purity of milk cannot be ascertained with certainty without an examination, which requires both apparatus and a good deal of chemical and bacteriological knowledge. In the ordinary daily examination of milk, however, comparatively simple tests are sufficient to ascertain whether the milk supplied varies greatly from the samples given before the contract was entered into. In most cases of falsification or adulteration, milk is watered or creamed, or submitted to both processes, and what is sold as new milk is often largely mixed with milk which has been skimmed. Now, watering alone can be detected at once by a lowering of the specific gravity and a diminished percentage of cream; while, when creaming alone is resorted to, the specific gravity is raised and of course the amount of cream is diminished. When, however, the milk is both creamed and watered the specific gravity may remain normal, so that the "dipper" of the lactometer does not by itself tell very much, and one has to practice to come back to the percentage of cream. After milk has been allowed to stand for from four to eight hours the cream should rise, the quantity which separates varying from 6 to 12 per cent., or even more. It is to be noted how very wide are the limits within which what is undoubtedly pure milk may vary in regard to richness of milk as stated in percentage of cream. This is the source of endless squabbling between dealers and public analyists, and the tendency of all dealers is to reduce the milk which they supply to the lowest standard which is accepted by the authorities as "pure milk." We think that the best way in which an institution can protect itself is to contract for "pure milk equal to sample," and to have the sample analysed by a responsible person, more especially as to the proportion of butter fat; the specific gravity and the amount of cream rising in a given number of hours being also noted for the guidance of the matron. It is then the simplest thing in the world for the matron to take the specific gravity and the amount of cream every day by the lactometer. On the occurrence of any material alteration of either one or the other she can warn the milkman that she has her eye on him, and, if he persists, a sample can be sent to the analyst. In this way all dispute as to the nature of the milk is avoided; it is a mere question whether it is up to date.—ED. *T. H.*

